# Peripheral Arterial Disease in the Context of Acute Coronary Syndrome: A Comprehensive Analysis of Its Influence on Ejection Fraction Deterioration and the Onset of Acute Heart Failure

**DOI:** 10.3390/jpm14030251

**Published:** 2024-02-26

**Authors:** Flavius-Alexandru Gherasie, Mihaela-Roxana Popescu, Alexandru Achim, Daniela Bartos

**Affiliations:** 1 Department of Cardiology, University of Medicine and Pharmacy “Carol Davila”, 050474 Bucharest, Romania; bartos_daniela@yahoo.co.uk; 2 Emergency Clinical Hospital Dr. Bagdasar-Arseni, 050474 Bucharest, Romania; 3 Elias University Emergency Hospital, 011461 Bucharest, Romania; 4 Nuffield Department of Population Health, University of Oxford, Oxford OX1 3UQ, UK; 5 Department of Cardiology, LKH-University Klinikum Graz Auenbruggerplatz 1, 8036 Graz, Austria; alexandru.achim@uniklinikum.kages.at; 6 Clinical University Emergency Hospital, 014461 Bucharest, Romania

**Keywords:** peripheral artery disease, ankle–brachial index, coronary artery disease, acute coronary syndrome, left ventricular ejection fraction, Killip class, HEART score

## Abstract

Background: Peripheral artery disease is a condition that causes narrowing of the arteries, impairing circulation to the extremities. Globally, it affects millions of people and is more prevalent in older adults and those with diabetes, high blood pressure, or high cholesterol. There is an overlap specific to polyvascular patients, and almost 50% of patients with PAD have coronary artery disease. Compelling evidence reveals a noteworthy association between PAD and major adverse cardiovascular events (MACEs) in individuals experiencing acute coronary syndrome (ACS) but limited knowledge exists regarding the influence of PAD on left ventricular systolic function during ACS. Methods: In a retrospective case–control study, we examined 100 participants who presented with ACS (mean age = 61.03 years, 80 [80%] males). The patients were divided into two groups: the ACS-PAD group (32 subjects, 74% of them with STEMI, 10% with NSTEMI, and 16% with NSTEACS) and the ACS-nonPAD group (68 participants). Results: This study highlighted that PAD negatively impacts patients with non-ST-segment elevation myocardial infarction (NSTEMI). These patients were likely to experience a decline of approximately 19.3% in their left ventricular ejection fraction (LVEF) compared to the ACS-nonPAD group (*p* = 0.003) and presented a worse clinical status (the PAD group correlated with Killip class IV, *p* = 0.049). Conclusion: Our analysis indicates that patients diagnosed with NSTEACS and PAD tend to have a higher LVEF of over 55% and a lower HEART score. Patients with PAD tend to have a functionally higher EF but clinically present with more unstable scenarios (pulmonary edema and cardiogenic shock). This is mainly driven by a higher prevalence of HFpEF in the PAD group. Looking closer at the PAD group, they have a higher incidence of comorbidities such as diabetes, hypertension, high cholesterol, CAD, and stroke, as well as being more active smokers.

## 1. Introduction

The prevalence and incidence of PAD are deeply affiliated with age, with an expansion of more than 10% affecting adults over 60 and 70. Given the global population’s aging, PAD is foreseen to become more prevalent. Men have a higher risk of experiencing the condition than women, particularly in cases of advanced disease [1].

Patients who suffer from peripheral arterial disease have decreased blood flow to their lower extremities. The condition is usually induced by atherosclerotic accumulations that narrow the vessel’s lumen and restrict the blood from reaching the distal extremity. This pathology can cause calf pain when walking due to inadequate blood flow and intermittent claudication. When a patient experiences rest pain, this may indicate a severe issue that requires immediate surgery to prevent further harm to the limb. Due to inadequate recognition, peripheral artery disease has been undiagnosed and poorly managed globally [2].

Lower-extremity peripheral artery disease generally refers to the hardening of the arteries that supply the limbs. This refers to arteries that run from iliac arteries to pedal arteries. This particular ailment is linked to negative clinical consequences, decreased physical capabilities, and limited physical activity. Despite its impact, it has received less attention and research than other atherosclerotic conditions like myocardial infarction. Over the past few years, considerable investigations have revealed that peripheral artery disease is directly connected with mortality, mainly as it raises the risk of future myocardial infarctions and strokes [3,4,5,6,7]. Numerous studies have shown that CAD and PAD often occur together; there seems to be a notable expansion in PAD occurrence in patients with coronary artery disease compared with those without it [8].

As previously discussed, individuals with peripheral arterial disease may encounter intermittent claudication. However, the manifestation of symptoms can vary in severity and is categorized by Fontaine’s scale [9].

The most commonly used non-invasive testing method relies on the ankle-to-brachial systolic blood pressure ratio, which needs a standard measurement procedure. The presence of PAD can be established by an ankle-to-brachial index ≤ 0.90 (ABI) [10]. A patient with peripheral artery disease is highly likely to have coronary artery disease. According to Kumar et al., the ABI can increase the pretest probabilities of CAD but cannot replace other testing methods [11]. In addition to ABI measurement, several methods exist for identifying peripheral artery disease. These methods include ultrasound evaluation, magnetic resonance angiography, computer tomographic angiography, and invasive angiography. Each method has its unique level of sensitivity and specificity [12,13].

When it comes to diagnosing acute coronary disease, physical examination findings alone may not be enough to determine if acute coronary syndromes are present. It is crucial to thoroughly evaluate the patient to assess their immediate risk, identify any mechanical complications associated with myocardial infarction, and recognize hemodynamic collapse. Suppose a patient is experiencing rapid heart rate, low blood pressure, and congestion signs such as pulmonary edema or hypoperfusion signs such as cool extremities. In that case, it is a sign of high clinical risk.

With the Killip classification, patients with STEMI and NSTEMI are graded from no signs of cardiac failure to cardiogenic shock, and this grading system is strongly representative of death rates [14].

Diagnosing acute coronary syndrome relies on a combination of clinical presentation, electrocardiogram (ECG) results, and biochemical evidence of myocardial injury. It is crucial to determine if a patient suspected of having acute coronary syndrome has ST-segment elevations on a 12-lead ECG or not [15,16].

The ECG and the evaluation of the function of the left ventricle come under the primary diagnostic procedure. All patients should go through a resting transthoracic echocardiogram to exclude other reasons for angina, identify regional wall motion abnormalities indicative of CAD, and assess LVEF for risk stratification [17,18].

As a clinical marker of cardiac function, ejection fraction is defined as the ratio between end-systolic volume and end-diastolic volume. Having a low LVEF is an indication of poor cardiac function and may indicate that further testing and treatment are recommended [19]. According to ejection fraction, we can categorize left ventricular dysfunction into severe LV dysfunction (LVEF < 40%), mild–moderate LV dysfunction (LVEF 41–49%), and preserved LV function (LVEF ≥ 50%) [20].

Over time, research papers have linked ejection fraction to morbidity and mortality, making it a hot discussion topic [21,22]. Perelshtein Brezinov et al. reported an increase in ACS admissions with preserved LVEF over a decade, while admission LVEF remains a strong predictor of 1-year mortality [21].

The HEART score was designed as a quick way to stratify patients with chest pain by their short-term risk of MACEs. It includes acute myocardial infarction, the need for percutaneous coronary intervention or bypass surgery, and the likelihood of death in the following six weeks. This scoring system aims to identify low-risk, moderate-risk, and high-risk patients [23]. The patient’s score is determined based on five variables: medical history, 12-lead ECG results, age, risk factors, and troponin levels. A score ranging from 0 to 2 is given in these five categories, with the highest possible score being 10 and the lowest possible score being 0. Patients with a score of 3 or less are considered low risk and have a MACE rate (major adverse cardiovascular events) of only 1.7%. These patients are safe for early discharge. However, if the score is higher, it can indicate an increased risk of MACEs and the need for further evaluation and intervention. Patients with a moderate score of 4–6 have a MACE rate of approximately 12–17% and may require observation and additional testing. If the score is high, between 7 and 10, the MACE rate is much higher (around 50–65%), and urgent or emergent intervention may be necessary [24,25].

The aim of this study is to assess if peripheral artery disease is a worsening factor for LVEF and acute heart failure in a population with acute coronary syndrome but without a history of peripheral artery disease before the index event (ACS). Our objective is to determine if there are differences between the three types of acute coronary syndromes (NSTEACS, STEMI, and NSTEMI) and ABI less than 0.9 in terms of LVEF prediction, Killip class at admission, mortality rates during hospital stays, and the necessity for urgent revascularization during hospital stays. This study’s results include admission periods; there is ongoing data collection for follow-up results.

## 2. Data and Methods

The present retrospective case–control study selected 100 participants who had presented with acute coronary syndromes to the Interventional Cardiology Unit of Elias University Hospital, Bucharest, Romania, between October 2019 and May 2022. The patients were divided into two groups: patients with ACS and with PAD (32 subjects) and patients without PAD (68 subjects).

The following inclusion criteria were used: patients presenting to Elias University Hospital with a diagnosis of one of the three types of ACS (NSTEACS, NSTEMI, and STEMI), written informed consent, patients over the age of 18.

Exclusion criteria comprised patients aged < 18, patients with previous vascular surgeries and angioplasties, and patients with a history of PAD (ankle–brachial index < 0.9 (ABI), carotid stenosis > 60%, femural artery stenosis > 50%).

To assess the relationship between variables, two distinct statistical models were employed. When the dependent variable took on continuous values, a linear regression model was utilized for estimation. Conversely, in instances where the dependent variable was binary, such as the occurrence or non-occurrence of an event, a logistic regression model was employed.

In both scenarios, the significance of the estimated beta parameter, which denotes the impact of the independent variable on the dependent variable, was evaluated using a *t*-test. A significance level (alpha) of 0.05 was adopted for this evaluation. Consequently, a *p*-value below 0.05 would be indicative of substantial evidence suggesting that the beta parameter significantly differs from zero, thereby establishing a relationship with the dependent variable.

The present study was carried out in line with the recommendations of the Helsinki Declaration and it was initiated after obtaining each patient’s informed consent as well as the approval of the Ethics Council of Elias University Hospital.

In this study, we used an ABI of less than 0.9 as a criterion for matching patients with PAD and an ABI of more than 0.9 for those without PAD.

Based on the fourth universal definition of myocardial infarction, the clinical definition of myocardial infarction defines it as the occurrence of acute myocardial injury alongside altered cardiac biomarkers levels (high cardiac troponin levels over the 99th percentile upper reference limit (URL) and occurrence of increased and/or decreased cardiac troponin levels) as the burden of proof for acute myocardial ischemia.

During the PCI procedures, the radial approach was used following classic techniques. A loading dose between 300 mg and 600 mg of clopidogrel and 250 mg of aspirin and intravenous unfractionated heparin at 70–100 IU/kg was provided to all patients. Following the intervention, patients were prescribed clopidogrel (75 mg daily) and aspirin (75–100 mg daily) for at least 12 months. All patients received third-generation drug-eluting stents.

The HEART score is a method to categorize patients as low-risk (0–3: possibly eligible for earlier dismissal), moderate-risk (4–6: potential nominee for additional assessment), or high-risk (7–10: likely candidates for immediate intervention).

The Killip classification approach is a clinical analysis tool for assessing patients with acute myocardial infarction. This method enables the determination of short-term and long-term consequences for patients with STEMI and NSTEMI and is suitable for guiding therapy strategies.

The Killip classes are divided into four categories, as follows: Killip class I: patients who do not show any signs or symptoms of heart failure; Killip class II: patients who exhibit crackles or rales in the lungs, increased jugular venous pressure, or an S3 gallop; Killip class III: patients who show signs of acute pulmonary edema; Killip class IV: patients who suffer from cardiogenic shock or hypotension (with systolic blood pressure below 90 mmHg) and display symptoms of low cardiac output, such as oliguria, cyanosis, or damaged mental status.

## 3. Statistical Analysis

Data processing was performed using the python 3.10 programming language and specific packages such as pandas and numpy. The statsmodels package was used to estimate the statistical models. The scipy package was used for the statistical tests.

## 4. Results

**Figure 1:**
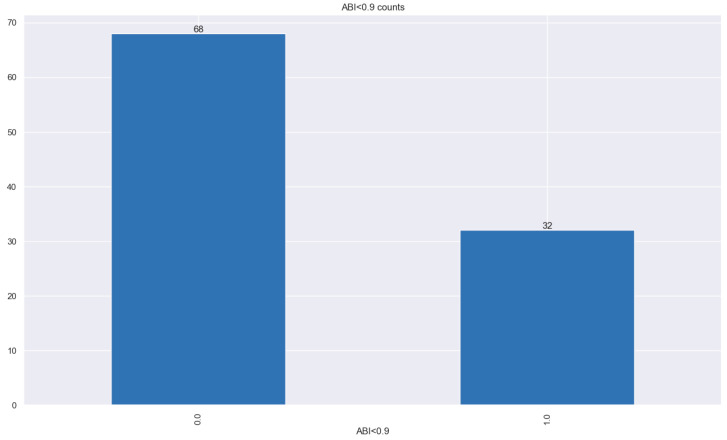
Baseline ABI < 0.9 in the study population. Over 0.9 on the left-hand side; less than 0.9 on the right-hand side.

**Figure 2:**
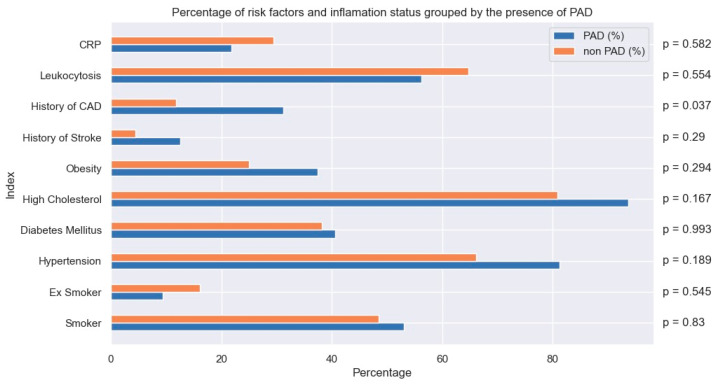
Percentage of risk factors and inflammation status grouped by the presence of PAD.

In this study, a total of 100 patients presenting with ACS (74% of them with STEMI, 10% with NSTEMI, and 16% with NSTEACS) who met our study criteria were included (mean age = 61.03 years, 80 [80%] males, 32 subjects with ABI < 0.9 (Figure 1)). The baseline clinical characteristics, LVEF levels, and Killip class at admission of the studied population are stratified in Table 1 and Figure 2.

**Figure 3:**
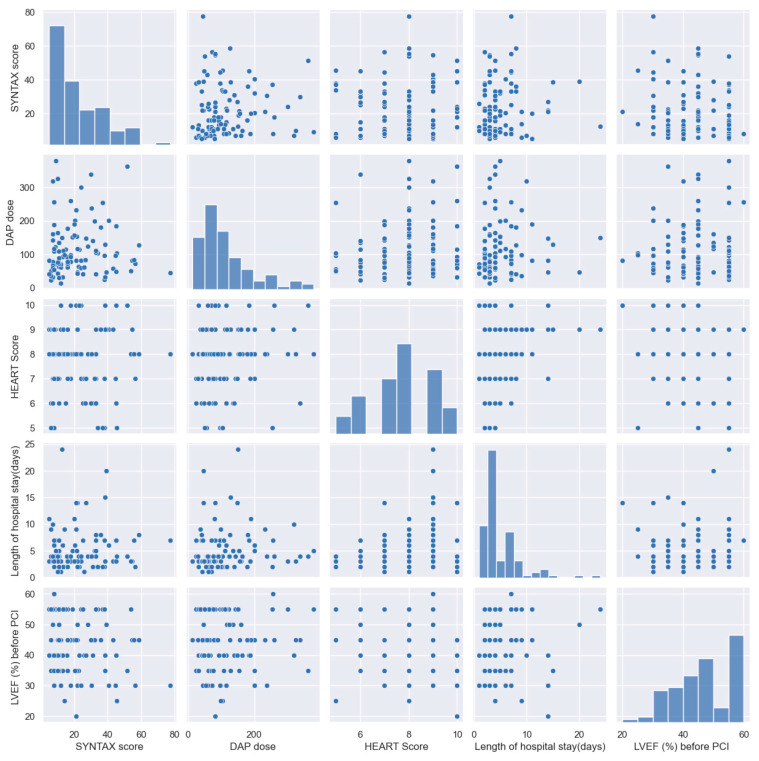
Distributions and scatter plots for continuous variables.

The study protocol included the evaluation of patients in terms of the presence of risk factors, age, presence of inflammation, the three types of ACS (STEACS, STEMI, and NSTEMI), ad hoc angiographic assessment of carotid arteries or lower limb arteries, ABI < 0.9, infarct-related artery, other lesions requiring revascularization, the SYNTAX score, other lesions stented during primary intervention, other lesions stented before discharge, other lesions stented after discharge, thromboaspiration during angioplasty, stent type, thrombolysis or lack thereof, presence of thrombolysis efficiency, dose–area product assessing radiation risk from diagnostic X-ray examinations (DAP dose), amount of contrast used, duration of the procedure, time from first chest pain until door-to-balloon, the HEART score, need for urgent revascularization during hospital stay, cardiac arrest during hospital stay, Killip class at admission, LVEF (%) before PCI, LVEF (%) after PCI and before discharge, and treatment before ACS (Table 2 and Table 3, Figure 3).

The distributions of the descriptive variables of the patients can be seen on the first diagonal in the image, and the scatterplots show the patients described by these variables, two by two. It can be observed that there are no outlier values among these variables. However, in the case of hospitalization days, there could be some patients with higher values, different from the others.

**Figure 4:**
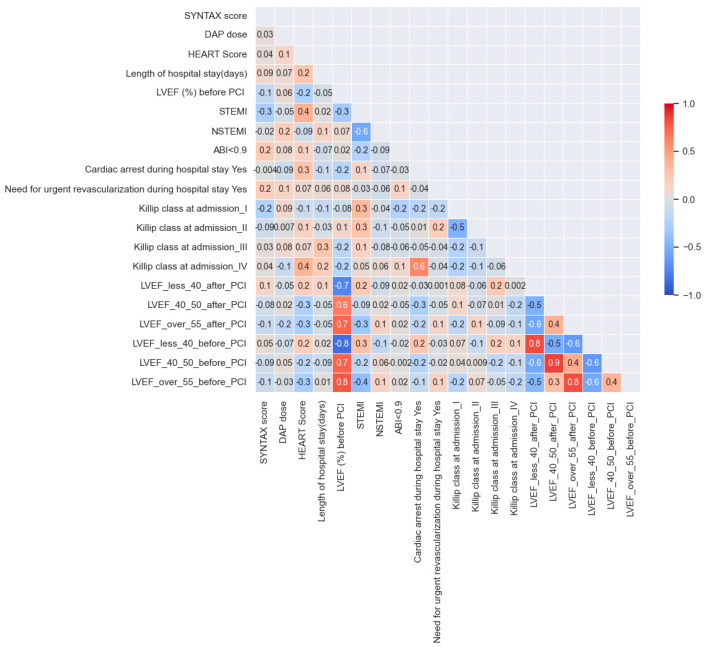
Pearson correlation matrix between variables.

In order to better understand the descriptive data of the patients in the sample, Pearson correlations were calculated and are represented in Figure 4. This also helped us intuitively predict which variables could be correlated with each other, thus requiring a more advanced analysis, such as a statistical test or model estimation. For example, HEART scores seem to be higher for STEMI patients (0.4 correlation). Another example would be SINTAX scores in patients with an ABI less than 0.9, where SINTAX scores tend to increase for patients with ABI less than 0.9, but in this case, it is a very small correlation, only 0.2. Even if some links are obvious, it remains interesting to measure the link between them in more detail.

### 4.1. Relationship between LVEF and PAD in Patients with NSTEACS

Our study discovered a correlation between LVEF higher than 55 before percutaneous coronary intervention (PCI) and ABI below 0.9 in patients with NSTEACS. We wanted to determine whether there is a significant relationship between the target variable, LVEF over 55, before PCI and patients with ABI below 0.9 and NSTEACS. In order to do that, a logistic regression was estimated (Table 4).

**Figure 5:**
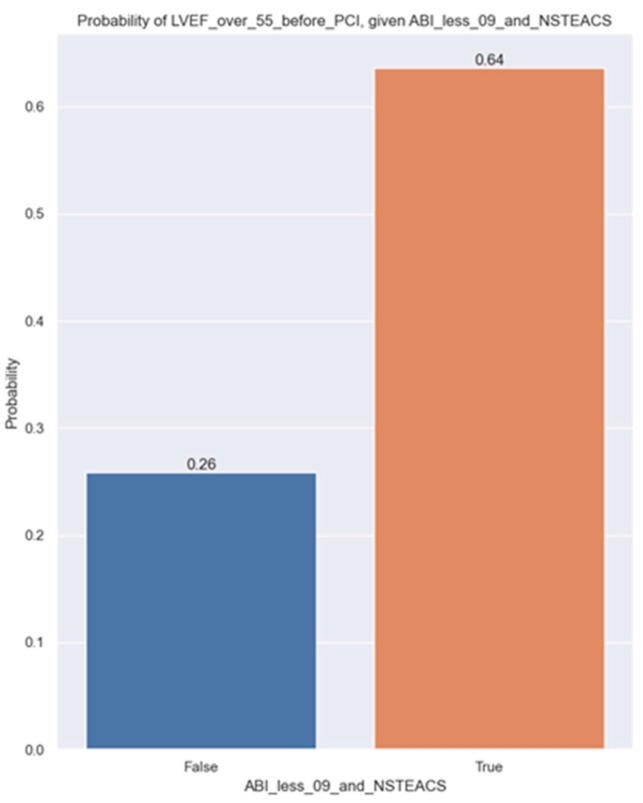
Probability of LVEF over 55% before PCI given ABI < 0.9 and NSTEACS.

As we can observe, the *p*-value is 0.016 and the estimation for the parameter beta1 is positive, so having an ABI less than 0.9 and NSTEACS increases the likelihood of having an LVEF over 55%. To have a better understanding of the probability of LVEF over 55, below are the computed probabilities for both cases, i.e., when a patient has an ABI under 09 and has NSTEACS and when they do not (Figure 5).

The estimated probability increases from 26% for a patient who does not have ABI under 0.9 and NSTEACS to 64% for a patient who meets these criteria.

**Figure 6:**
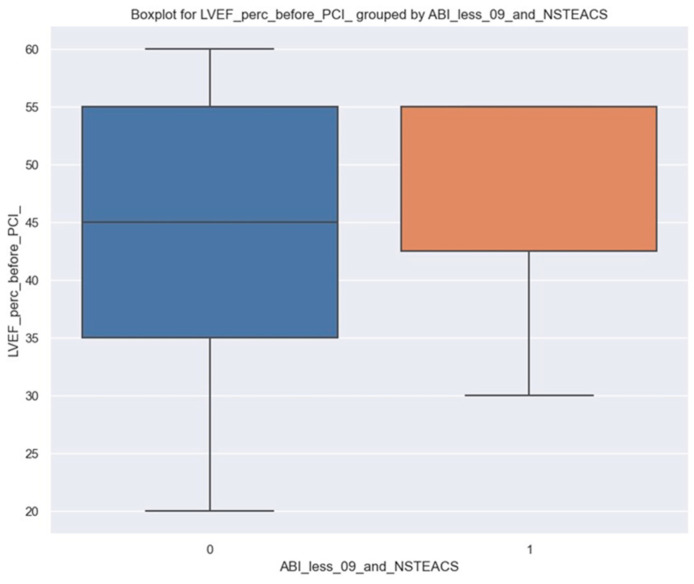
Boxplot for LVEF percentage before PCI; left side, blue column: LVEF percentage before PCI in patients without ABI less than 0.9 and NSTEACS; right side, orange column: LVEF percentage before PCI in patients with ABI less than 0.9 and NSTEACS.

If we look only at the distribution of LVEF grouped by each type of patient, we will see a difference here as well. It is observed that there is a slight difference for those with ABI less than 0.9 and NSTEACS; it is slightly higher than for others, 49 vs. 43.3 (Figure 6).

It is essential to test whether or not these differences are random and how significant they might be at a population level. For that, a linear regression was fitted (Table 5).

In this case, ABI less than 0.9 and NSTEACS do not seem to have a very clear impact on LVEF; the *p*-value is 0.056, very close to 0.05. Even though it is likely there is an impact of LVEF, other studies with more data would be indicated. Based on our sample, patients with ABI under 0.9 and NSTEACS tend to have, on average, LVEF 5.7% greater than others. However, the 95% confidence interval is between −0.1% and 11.6%, so there are some chances that this impact does not actually exist at the population level.

### 4.2. Relationship between LVEF and PAD in Patients with NSTEMI

**Figure 7:**
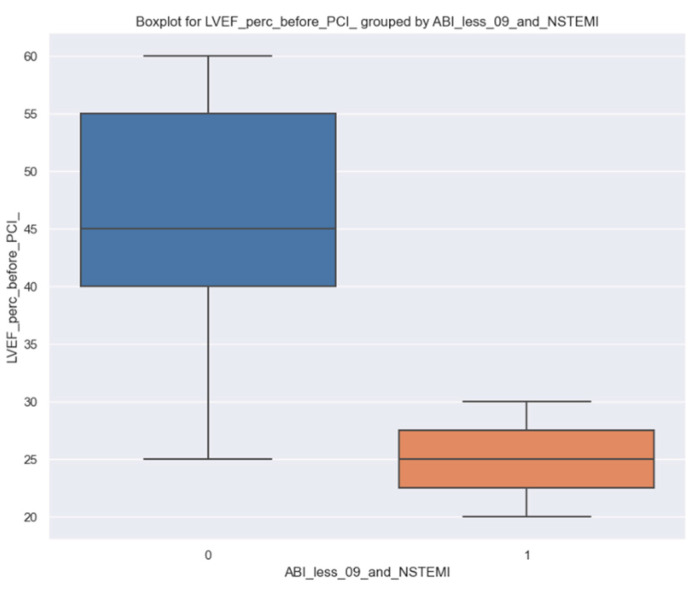
Boxplot for LVEF percentage before PCI; left side, blue column: LVEF percentage before PCI in patients without ABI less than 0.9 and NSTEMI; right side, orange column: LVEF percentage before PCI in patients with ABI less than 0.9 and NSTEMI.

In the group of patients with NSTEMI and ABI less than 0.9, we used the LVEF value as the target variable and ABI less than 0.9 as the independent variable for NSTEMI. We intended to examine how the status of a patient affects their LVEF (Figure 7).

It can be seen that there is a difference in LVEF between these two groups of patients, i.e., the ones who have ABI less 0.9 and NSTEMI and those who do not. It is worth mentioning that we have a highly unbalanced proportion of patients; those with ABI less than 0.9 and NSTEMI are less frequent.

In order to obtain a more precise impact of this status of patients on LVEF, linear regression parameters were estimated (Table 6).

Here are some comments regarding the output of linear regression. Only 8.5% of the variance in LVEF is explained by the status of a patient having ABI less 0.9 and NSTEMI. If a patient has an ABI under 0.9 and NSTEMI, on average, LVEF decreases by 19.3%, but this is just the best estimation given our sample; at the population level, this impact could be a decrease between 32.1% and 6.5% with a 95% confidence interval, so we are confident enough that the effect of our variable is not equal to 0 (*p*-value 0.003).

### 4.3. How Does Peripheral Artery Disease Impact Killip Class in Patients with Acute Myocardial Infarction?

Upon analyzing Killip class at admission, we discovered a correlation between Killip class I and ABI less than 0.9. The target variable used in the logistic regression is whether or not a patient is in Killip class I. The independent variable is whether or not a patient has an ABI less than 0.9. In this case, if a patient has an ABI less than 0.9, then the probability of them being Killip class I tends to decrease, and we are confident that this is true at the population level (*p*-value = 0.016). Even though an ABI of less than 0.9 does not explain much of the variance in Killip class I, it still has an impact, and the pseudo-R-squared value explains this (Table 7).

**Figure 8:**
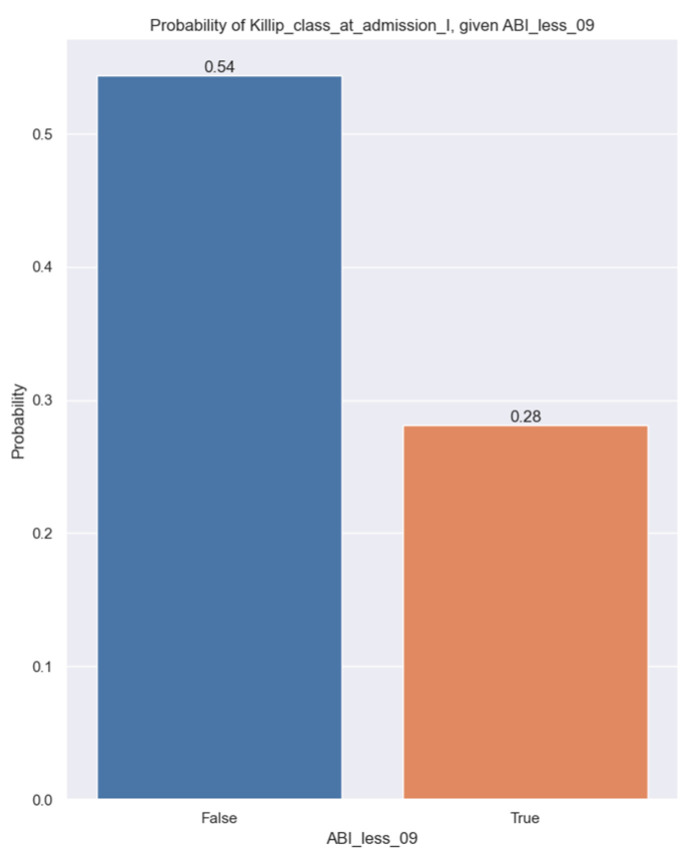
Probability of Killip class I at admission given ABI less than 0.9.

The following figure shows how the probabilities change based on our independent variable, ABI less than 0.9. So, if a patient has an ABI under 09, their chances of being in Killip class I decrease from 54% to 28% (Figure 8).

Our study found a significant correlation between Killip class IV and ABI less than 0.9 in patients with NSTEMI. The logistic regression estimate explains how the probability of being in Killip class IV is affected for patients with ABI less than 0.9 and NSTEMI (Table 8).

**Figure 9:**
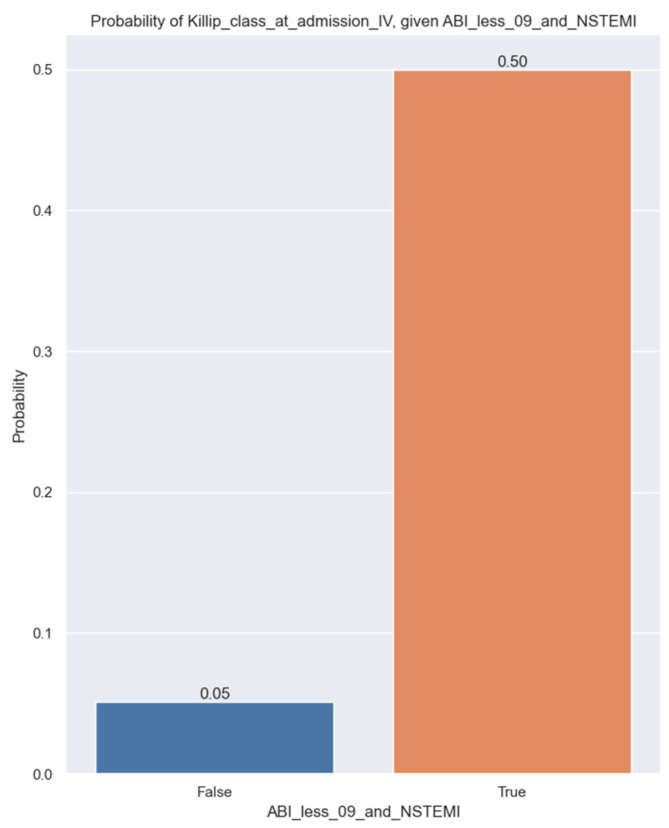
Probability of Killip class IV at admission given ABI less than 0.9 and NSTEMI.

The coefficient for ABI less than 0.9 and NSTEMI is positive, and the associated *p*-value is 0.049. Since it is not close to 0, we still have some doubts about whether it has an impact. beta1 on the population level is between 0.009 and 5.83, so it is likely to have an effect on the probability of a patient being Killip IV given ABI under 09 and NSTEMI status. The estimated value of the beta1 parameter is 2.92, and the below plot shows how this affects the probability (Figure 9).

### 4.4. Correlation between HEART Score and PAD in ACS Patients

**Figure 10:**
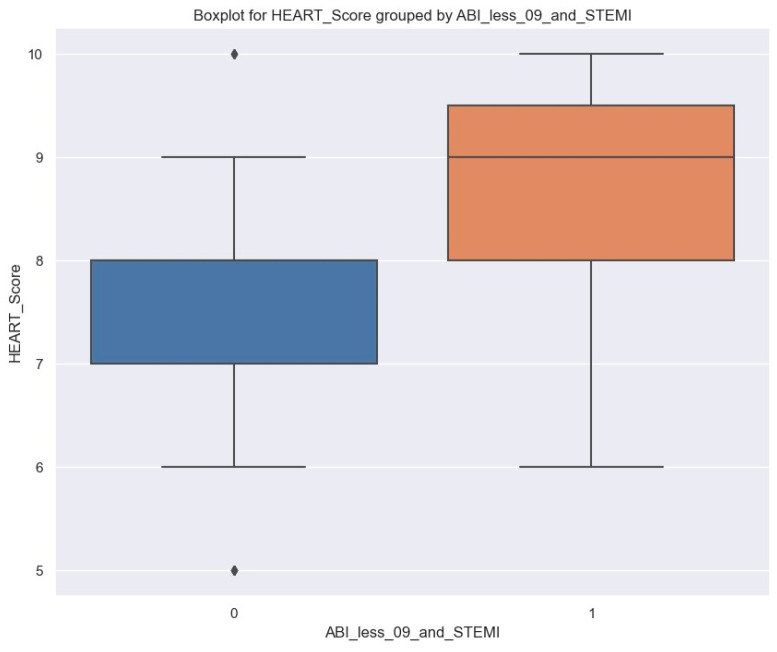
Boxplot of HEART score grouped by ABI less than 0.9 and STEMI; left side, blue column: patients without ABI less than 0.9 and STEMI; right side, orange column: patients with ABI less than 0.9 and STEMI.

To explain the correlation between HEART score, ABI less than 0.9, and STEMI patients, we can observe the distribution of HEART score in these two groups—patients with ABI less than 0.9 and STEMI and others. There is a noticeable difference between the two groups (Figure 10).

Patients with ABI less than 0.9 and STEMI tend to have higher HEART scores. A linear regression was estimated to find how HEART score is affected for patients with ABI less than 0.9 and STEMI (Table 9).

We are confident that ABI less than 0.9 and STEMI are associated with a higher HEART score. On average, this score increases by 1.06. At the population level, it could rise from 0.41 to 1.71 with 95% confidence. A proportion of 10%; of the variance in HEART score is explained by the status of a patient having an ABI less than 0.9 and STEMI; other factors like diet, lifestyle, etc., define the remaining 90%.

**Figure 11:**
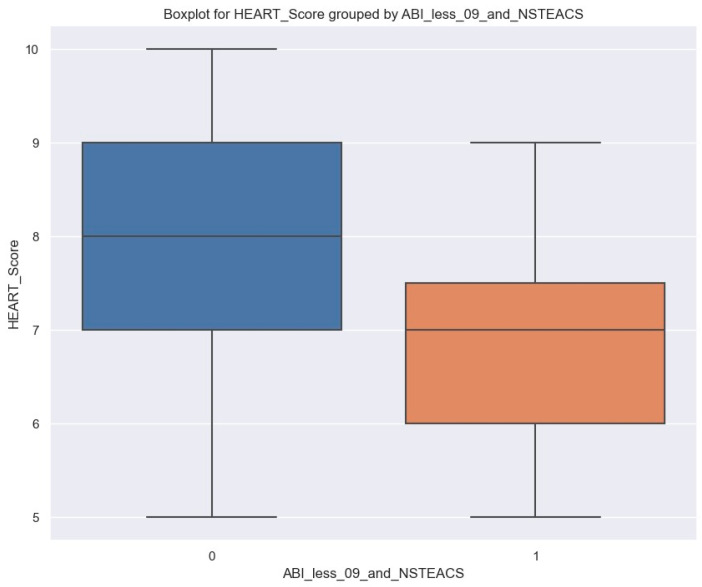
Boxplot of HEART Score grouped by ABI less than 0.9 and NSTEACS; left side, blue column: patients without ABI less than 0.9 and NSTEACS; right side, orange column: patients with ABI less than 0.9 and NSTEACS.

It is important to note that individuals with an ABI less than 0.9 and NSTEACS may have a lower HEART score (Figure 11).

In order to measure how much this status is associated with HEART score, a linear regression was fitted (Table 10).

On average, patients with ABI less than 0.9 and NSTEACS have HEART scores lower by 0.96, but this value applies to our sample; at the population level, it is between 1.79 and 0.136 with 95% confidence.

There were four cardiac deaths during hospital stay. One of the deceased patients had an ABI of less than 0.9. There was no need for urgent revascularization for any patients with acute coronary syndrome and an ABI of less than 0.9.

To facilitate comprehensive analysis, Table 11 consolidates key information from all preceding regressions, ensuring ease of comparison across the results.

## 5. Discussion

While an ABI of less than 0.9 is commonly used as a diagnostic tool for peripheral artery disease, recent studies have suggested that it is also a valuable marker for predicting mortality and prognosis in patients with more than one arterial territory disease, such as those with CAD [26,27,28]. Because this study aimed to evaluate the LVEF of ACS patients with ABI less than 0.9, it revealed new information about this pathology. After reviewing the relevant literature, we found no studies on the LVEF of ACS patients with ABI less than 0.9.

According to Rantner et al., PAD patients display substantially lower LVEF levels than those without PAD. The percentage of patients with LVEF levels below 55% in PAD patients was 30%, compared to 7% in controls (*p* < 0.001) [29]. Our study indicates that individuals with an ABI lower than 0.9 and NSTEMI are more likely to have an LVEF greater than 55%, with a *p*-value of 0.016. Our research also suggests that individuals with an ABI lower than 0.9 and NSTEACS tend to have, on average, LVEF 5.7% greater than others. However, the *p*-value for this observation is 0.056, which is very close to 0.05. Therefore, further studies with more data would be required to confirm this finding. Patients diagnosed with NSTEACS may typically be older, with a mean age exceeding 60 [30,31]. As a result, more of these patients may suffer from chronic coronary disease, which may help the culprit artery and bring blood flow through the donor vessel. This mechanism could increase the chances of preserving LVEF, recovering LVEF, and reducing the myocardial infarction area and myocardial scarring [2].

Suppose a patient has an ABI of less than 0.9 and is experiencing NSTEMI. According to our study, their LVEF is expected to decline by approximately 19.3% (*p*-value = 0.003). However, it is essential to note that this estimation is based on a limited sample size and may not represent all patients. Additionally, only 8.5% of the patient population’s characteristics can explain the variance in LVEF.

Our study discovered that patients with an ABI less than 0.9 are less likely to be in Killip class I (*p*-value = 0.016). In addition, patients with an ABI less than 0.9 and NSTEMI are more likely to be in Killip class IV, with a *p*-value of 0.049. The present study revealed that PAD has a detrimental effect on patients who suffer from NSTEMI. These patients are expected to face a decline of around 19.3% in their LVEF and are more likely to be classified in Killip class IV. Moreover, they may encounter worse in-hospital outcomes, such as an increased death rate and heart failure [32]. This agrees with the results of the CRUSADE registry, in which PAD was an independent indicator of heart failure in NSTEMI patients [33,34,35].

Patients with an ABI of less than 0.9 and diagnosed with STEMI typically exhibit higher HEART scores. On average, such patients’ HEART scores increase by 1.06 (*p* = 0.002). However, patients with an ABI of less than 0.9 and NSTEACS may have lower HEART scores. On average, these patients’ HEART scores decrease by 0.96 (*p* = 0.023). Overall, we can conclude that NSTEACS patients with peripheral artery disease are more likely to have an LVEF of over 55% and a lower HEART score than those without, which can positively impact their admission and short-term follow-up.

## 6. Conclusions

The study found that patients with peripheral artery disease presenting with acute coronary syndrome tend to have a higher ejection fraction but clinically present with more unstable scenarios, such as pulmonary edema and cardiogenic shock. The presence of peripheral artery disease may be a helpful tool for classifying the risk of ejection fraction depression before revascularization based on the type of ACS.

## Figures and Tables

**Table 1:** Baseline characteristics, LVEF levels, and Killip class at admission.

Binary Variable	Counts (N = 100)
STEMI	74
NSTEMI	10
NSTEACS	16
ABI < 0.9	32
Cardiac arrest during hospital stay (yes)	4
Need for urgent revascularization during hospital stay (yes)	3
Killip class at admission_I	46
Killip class at admission_II	22
Killip class at admission_III	5
Killip class at admission_IV	6
LVEF_less_40_after_PCI	33
LVEF_40_50_after_PCI	78
LVEF_over_55_after_PCI	39
LVEF_less_40_before_PCI	42
LVEF_40_50_before_PCI	72
LVEF_over_55_before_PCI	30

**Table 2:** Summary statistics for continuous variables.

	SYNTAX Score	DAP Dose	HEART Score	Length of Hospital Stay (Days)	LVEF (%) before PCI
count	100	99	100	99	99
mean	22.3	115.9	7.8	5.1	44
std	15.1	79.8	1.3	3.8	9.4
min	5	14	5	1	20
25%	10.8	61.5	7	3	35
50%	18	93	8	4	45
75%	32.2	149	9	7	55
max	77.5	379	10	24	60

**Table 3:** Counts of binary variables.

Binary Variable	Counts (N = 100)
STEMI	74
NSTEMI	10
NSTEACS	16
ABI < 0.9	32
Cardiac arrest during hospital stay (yes)	4
Need for urgent revascularization during hospital stay (yes)	3
Killip class at admission_I	46
Killip class at admission_II	22
Killip class at admission_III	5
Killip class at admission_IV	6

**Table 4:** Logistic regression of LVEF over 55 before PCI~ABI less than 09 and NSTEACS output.

Dep. Variable:	LVEF_over_55_before_PCI	No. Observations:	100			
Model:	Logit	Df Residuals:	98			
Method:	MLE	Df Model:	1			
Date:	Sun, 23 July 2023	Pseudo R-squ.:	0.04946			
converged:	TRUE	LL-Null:	−61.086			
	coef	std err	z	P > |z|	[0.025	0.975]
Intercept	−1.0542	0.242	−4.354	0	−1.529	−0.58
ABI_less_09_and_NSTEACS	1.6138	0.672	2.402	0.016	0.297	2.931

**Table 5:** Linear regression of LVEF percentage before PCI~ABI less than 09 and NSTEACS output.

Dep. Variable:	LVEF_perc_before_PCI_	R-Squared:	0.037			
Model:	OLS	Adj. R-squared:	0.027			
Method:	Least Squares	F-statistic:	3.751			
Date:	Sun, 23 July 2023	Prob (F-statistic):	0.0557			
No. Observations:	99	AIC:	723.7			
	coef	std err	t	P > |t|	[0.025	0.975]
Intercept	43.3523	0.988	43.893	0	41.392	45.313
ABI_less_09_and_NSTEACS	5.7386	2.963	1.937	0.056	−0.142	11.619

**Table 6:** Linear regression of LVEF percentage before PCI~ABI less than 0.9 and NSTEMI output.

Dep. Variable:	LVEF_perc_before_PCI_	R-Squared:	0.085			
Model:	OLS	Adj. R-squared:	0.076			
Method:	Least Squares	F-statistic:	9.024			
Date:	Sun, 23 July 2023	Prob (F-statistic):	0.00339			
No. Observations:	99	AIC:	718.7			
	coef	std err	t	P > |t|	[0.025	0.975]
Intercept	44.3814	0.917	48.396	0	42.561	46.202
ABI_less_09_and_NSTEMI	−19.3814	6.452	−3.004	0.003	−32.187	−6.576

**Table 7:** Logistic regression of Killip class at admission I~ABI less than 09 output.

Dep. Variable:	Killip_class_at_admission_I	No. Observations:	100			
Model:	Logit	Df Residuals:	98			
Method:	MLE	Df Model:	1			
Date:	Sun, 23 July 2023	Pseudo R-squ.:	0.04512			
converged:	TRUE	LL-Null:	−68.994			
	coef	std err	z	P > |z|	[0.025	0.975]
Intercept	0.1769	0.243	0.727	0.467	−0.3	0.654
ABI_less_09	−1.1152	0.462	−2.411	0.016	−2.022	−0.209

**Table 8:** Logistic regression of Killip class at admision IV~ABI less than 09 and NSTEMI.

Dep. Variable:	Killip_class_at_admission_IV	No. Observations:	100			
Model:	Logit	Df Residuals:	98			
Method:	MLE	Df Model:	1			
Date:	Sun, 23 July 2023	Pseudo R-squ.:	0.06885			
converged:	TRUE	LL-Null:	−22.697			
	coef	std err	z	P > |z|	[0.025	0.975]
Intercept	−2.9232	0.459	−6.367	0	−3.823	−2.023
ABI_less_09_and_NSTEMI	2.9232	1.487	1.966	0.049	0.009	5.837

**Table 9:** Linear regression of HEART score~ABI less than 09 and STEMI.

Dep. Variable:	HEART_Score	R-Squared:	0.098			
Model:	OLS	Adj. R-squared:	0.089			
Method:	Least Squares	F-statistic:	10.65			
Date:	Sun, 23 July 2023	Prob (F-statistic):	0.00152			
No. Observations:	100	AIC:	335			
	coef	std err	t	P > |t|	[0.025	0.975]
Intercept	7.5679	0.142	53.256	0	7.286	7.85
ABI_less_09_and_STEMI	1.0637	0.326	3.263	0.002	0.417	1.711

**Table 10:** Linear regression of HEART score~ABI less than 09 and NSTEACS.

Dep. Variable:	HEART_Score	R-Squared:	0.052			
Model:	OLS	Adj. R-squared:	0.042			
Method:	Least Squares	F-statistic:	5.326			
Date:	Sun, 23 July 2023	Prob (F-statistic):	0.0231			
No. Observations:	100	AIC:	340			
	coef	std err	t	P > |t|	[0.025	0.975]
Intercept	7.8764	0.139	56.659	0	7.601	8.152
ABI_less_09_and_NSTEACS	−0.9673	0.419	−2.308	0.023	−1.799	−0.136

**Table 11:** Summary of all regressions fitted.

Independent Variable	Dependent Variable	Type of Independent Variable	Regression Type	Beta 0 Coef	Beta 1 Coef	*p*-Value Coef Beta 1
ABI_less_09_and_NSTEACS	LVEF_over_55_before_PCI	binary	logistic	−1.0542	1.6138	0.016
ABI_less_09_and_NSTEACS	LVEF_perc_before_PCI_	continuous	linear	43.3523	5.7386	0.056
ABI_less_09_and_NSTEMI	LVEF_perc_before_PCI_	continuous	linear	−19.3814	6.452	0.003
ABI_less_09	Killip_class_at_admission_I	binary	logistic	0.1769	−1.1152	0.016
ABI_less_09_and_NSTEMI	Killip_class_at_admission_IV	binary	logistic	−2.9232	2.9232	0.049
ABI_less_09_and_STEMI	HEART_Score	continuous	linear	7.5679	1.0637	0.002
ABI_less_09_and_NSTEACS	HEART_Score	continuous	linear	7.8764	−0.9673	0.023
MPV	Preserved LVEF	binary	logistic	5.4	−0.58	0.001

## Data Availability

The data analyzed during the current study are available from the corresponding author upon reasonable request.
